# Do Individual and Neighborhood Characteristics Influence Perceived Air Quality?

**DOI:** 10.3390/ijerph14121559

**Published:** 2017-12-12

**Authors:** Séverine Deguen, Manon Padilla, Cindy Padilla, Wahida Kihal-Talantikite

**Affiliations:** 1 EHESP School of Public Health, 35043 Rennes CEDEX, France; manonpadilla@gmail.com (M.P.); cindy.padilla@ehesp.fr (C.P.); 2 Department of Social Epidemiology, Institut Pierre Louis d’Epidémiologie et de Santé Publique (UMRS 1136), INSERM, Sorbonne Universités, Université Pierre et Marie Curie, 75012 Paris, France; 3 Laboratoire Image Ville Environnement (LIVE) UMR 7362 CNRS, University of Strasbourg, 6700 Strasbourg, France; wahida.kihal@live-cnrs.unistra.fr

**Keywords:** air pollution, perception, public health

## Abstract

*Background*: Despite improvements, air pollution still remains a major public health issue. Numerous epidemiological studies have demonstrated the adverse health effects of air pollution exposure based on modeled measures, but only a few have considered the health impact of perceived air quality. Improving our knowledge of individual perceptions is crucial to defining targeted actions and promoting appropriate intervention measures. Our objective is to investigate the relationship between subjective and objective measures of air pollution and to focus on how individual characteristics combined with the neighborhood socioeconomic deprivation index, measured at a fine spatial scale, may or may not alter this relationship. *Materials and Methods*: The subjective measures of air quality reported by a sample of Lyon residents were collected via an individual questionnaire. The objective measures of air pollution were modeled by the local air quality monitoring network of the Rhône-Alpes region at census block level. We used a socioeconomic deprivation index to capture the different socioeconomic dimensions at census block level. The statistical analysis was structured in two steps: (1) identification of individual determinants of the subjective measures of air quality using multiple correspondence analysis followed by hierarchical clustering; (2) identification of individual and contextual characteristics that may alter the relationship between the objective and subjective measures of air pollution. *Results*: Among the youngest and the middle aged population (ages 30 to 59), consistent results between level of satisfaction, perceived air quality and objective measures of air pollution were found whatever the individual characteristics of the population. It is less clear among the oldest population: globally no significant difference between the NO_2_ concentrations and the level of satisfaction was observed. *Conclusions*: We found a significant relationship between the subjective and objective measures of air pollution in many population sub-groups with different combinations of individual characteristics. The relationship is less clear among the oldest population, which confirms previous findings. Our finding highlights that age combined with low level of education and unemployment, or women or health problems as well as the neighborhood deprivation index influence the level of air quality satisfaction.

## 1. Introduction

Despite improvements in air quality in most developed countries, air pollution remains a major environmental and public health issue affecting everyone in developed and developing countries alike [1]. Numerous studies have demonstrated adverse health effects of air pollution exposure, including chronic and acute diseases and mortality. They reported associations with long term exposure to particulate air pollution and nitrogen dioxide [2,3,4,5,6] as well as with short term exposure [7,8]. Most of the studies have based their analysis on objective measures of air pollution resulting from different methods such as those based on geospatial techniques to estimate the proximity to high traffic roads [9] or monitoring stations [10,11,12], and those based on modeling to estimate air pollutant concentrations at a given statistical unit [13]. To a lesser extent, other epidemiological studies used individual sensors to obtain a better measurement of the exposure to air pollution. 

Today, there are more and more experimental and epidemiological studies suggesting a neurobehavioral effect of air pollution exposure [14,15,16,17]. These studies revealed symptoms of anxiety and depression [17,18,19] or psychiatric emergencies [20] among children as well as adults [16,18] associated with exposure to air pollution. In addition, some studies hypothesize that the association between air pollution and depression may be mediated by perceived air quality [21,22].

Among the research focusing on individual perception of air pollution, studies investigating relationships between subjective measures (perception) and objective measures (quantitative measures) of air pollution revealed inconsistent results. Some studies found that people were not aware of high air pollution levels in their residential area [23,24,25], whereas others showed a significant correlation between awareness of air pollution and the concentration of some air pollutants measured at monitoring stations [26,27]. One hypothesis that could explain these inconsistent results is the heterogeneity and variability of individual characteristics including socioeconomic and demographic factors (age and gender, for instance] [27,28,29,30,31]. In spite of these contrasted findings reported in the literature regarding the relationship between perception scores and measures of air pollution, there is a consensus that perception does play a role in public response to environmental exposures [26,32]. 

Health status, including self-reported health problems may modify the perceived environmental quality and then may influence the response and acceptance of actions or interventions aimed at improving air quality [33]. Today, the strategy of reducing air pollution and the associated health impacts is based on both community and individual levels and implies that “*community and individual’s perceptions on exposure are critical in determining people’s response and acceptance of related policies*” [33]. Therefore, improving our understanding and knowledge of the perceptions of both exposure and associated health risk is crucial to designing targeted strategies and guiding policymakers in defining and promoting appropriate intervention actions. To maximize the chances of success of preventive measures and to adapt them if necessary, it appears important to consider people’s perceptions. 

Less attention is paid to the feelings and the subjective perceptions of air pollution in the general population, which calls for additional research to fully assess the health impact of air pollution based on the premise that air pollution may cause both physical and psychological harm. 

In this context, the main objective of our study is to investigate the relationship between the subjective measures of air pollution reported by a sample of Lyon residents and objective measures of air pollution modeled by the local air quality monitoring network. In addition, the study will focus on how individual characteristics combined with the neighborhood socioeconomic deprivation index, measured at a fine spatial scale, may or may not alter this relationship.

## 2. Materials and Methods

### 2.1. Study Setting and Statistical Units

The study was carried out in the Lyon metropolitan area (LMA), one of the largest urban areas located in the Rhône-Alpes region of central-eastern France. The total population is approximately 1.2 million inhabitants across an area of 527 km^2^. Our study area was sub-divided into 56 municipalities and 499 census blocks (called IRIS by INSEE, the French National Statistics Institute). These units are constructed so as to be as homogeneous as possible in terms of population size. In the LMA, census blocks ranged from 0 to about 7200 residents with an average of approximately 2500 residents.

### 2.2. Subjective Measures of Air Pollution and Individual Characteristics

The subjective measures of air quality were collected via a questionnaire administered by phone in May 2014. A preliminary study was conducted in order to test the acceptability of the study by the inhabitant of the Lyon Metropolitan area and to improve the formulation of the questions. The procedure used to select the study population was structured in several successive steps:

- Census blocks of LMA were first classified according to the median level of nitrogen dioxide (NO_2_ expressed in μg/m^3^) and noise level (equal to 40.2 μg/m^3^ and 32.9 decibels, respectively). Next, four distinct categories of census blocks were constructed: census blocks with: (i) lower levels of NO_2_ and noise than the median; (ii) a lower level of NO_2_ and a higher level of noise than the median; (iii) a higher level of NO_2_ and a lower level of noise than the median, and (iv) higher levels of NO_2_ and noise than the median. Noise and air pollution indicators were chosen as good indicators of environmental pollution in the LMA. Indeed, in the Lyon Metropolitan Area, traffic road has been recognized to be one of the main contributors of air pollution (http://www.ppa-lyon.org/). Noise exposure constitutes additional environmental consequences of the traffic road. In addition, following the recommendation of the second plan of environmental health of the Rhône-Alpes region, a web platform named Orphane (http://www.orhane.fr/) has been created to identify the part of the region accumulating high levels of air pollution and noise to better target action and decision making.
- Next, 2500 phone numbers per category were randomly selected from the France Telecom Institute and other phone companies—creating a random data base of 10,000 phone numbers.
- Finally, in each category, phone numbers were called until 250 people answered. Three calls, on different days and at different times, were made before moving to another phone number. In total, the study population included 1000 people.

The questionnaire was organized into two parts; the first part contained questions dealing with socioeconomics (level of education, occupational situation, job, marital status), demographics (gender and age) and housing characteristics, means of travel to work and health status (self-reported respiratory and cardiovascular problems);the second part dealt with the perception of air quality, knowledge about interventions aimed at improving air quality and the level of satisfaction with the place of residence. The four questions are listed below:

(1) Do you agree with the following statement: “the quality of the air you breathe within your residential census block is good”
  $$\text{Three possible answers}\;\{\begin{array}{c} Yes\;I\;totally\;agree\\ \;I\;somewhat\;agree\\ I\;do\;not\;agree \end{array}$$
(2) Would you ever consider living in another place where air quality is better? (Yes/no)
(3) Are you aware of any actions or interventions aimed at improving air quality in the Lyon Metropolitan area? (Yes/no)
(4) Would you say that you are satisfied with the place where you live?
  $$\text{Three possible answers}\;\begin{array}{c} Yes\;I\;totally\;agree\\ I\;somewhat\;agree\\ I\;do\;not\;agree \end{array}$$

The postal addresses of each individual were geocoded at the census block by the Geocible Institute (www.geocible.fr).

### 2.3. Objective Measures of Ambient Air Pollution and Neighborhood Socioeconomic Index

Four indicators were chosen to characterize the quality of the environment (named objective measures of air pollution): air pollution, heavy residential traffic exposure, noise exposure and green space exposure [34].

#### 2.3.1. Air Pollution

Annual average ambient concentrations of nitrogen dioxide (NO_2_ expressed in μg/m^3^) were modeled at a 10 × 10 m^2^ resolution and then aggregated at the census block level by the local air quality monitoring network of the Rhone-Alpes region (Atmo Rhone Alpes) over the period 2002 to 2012. A determinist model called SIRANE was used, including different input parameters such as meteorological data, emission sources and background pollution measurements. (More methodological details about estimated NO_2_ data are available elsewhere [13]). NO_2_ is known to be a good tracer of pollution generated by traffic and other combustion sources (main sources of pollution in Lyon metropolitan area). This pollutant has also been recognized to have a greater spatial variability compared to the PM_10_ or PM_2.5_.

**Figure 1:**
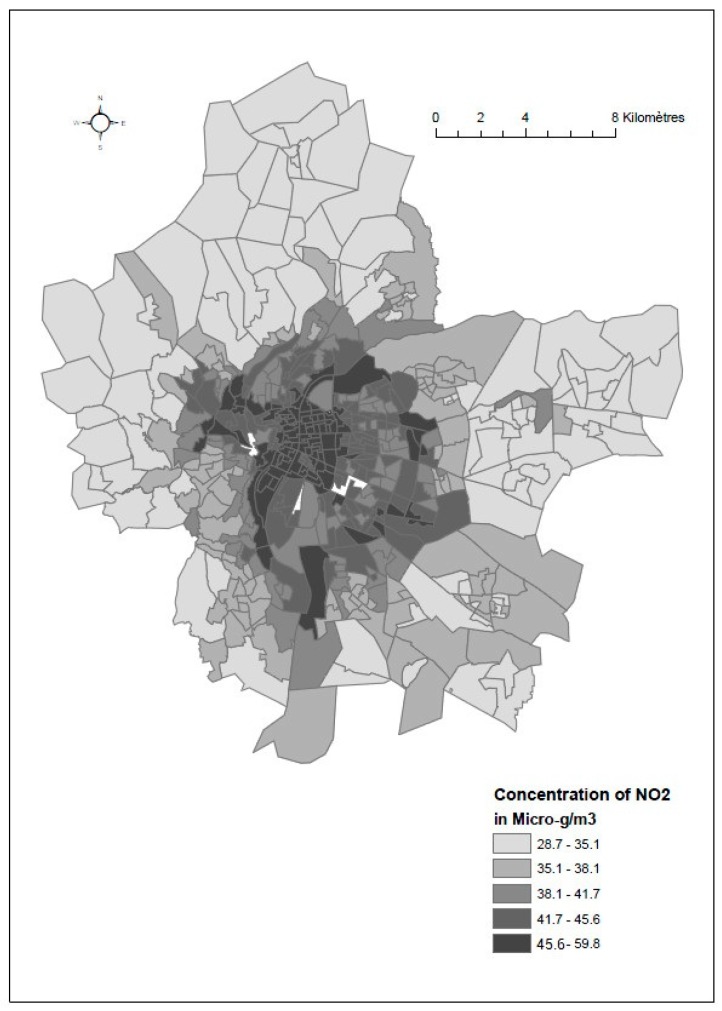
Spatial distribution of annual average NO_2_ exposure over the period 2002–2012, at census block level based on quintiles of the distribution.

Figure 1 represents the spatial distribution of annual average NO_2_ concentrations over the period 2002 to 2012. It shows a clear gradient from the north- eastern with lower NO_2_ concentrations, to the south-west with higher NO_2_ concentrations.

#### 2.3.2. Heavy Residential Traffic Exposure

Heavy residential traffic exposure was measured by computing the percentage of inhabitants in each census block living within a specified distance from the road [35,36]. The local air quality monitoring network (Atmo Air Rhône-Alpes) provided the traffic density data and a spatial dataset for roads and highways in the Lyon Metropolitan Area in 2005. More precisely, this dataset included Annual Average Daily Traffic (AADT) as an attribute describing traffic volume (provided by the Greater Lyon Urban Community) for each road (the number of vehicles per 24-h period on all roads in the study area). In our study, major roads were defined as those that had an AADT higher than 5000 vehicles per day.

**Figure 2:**
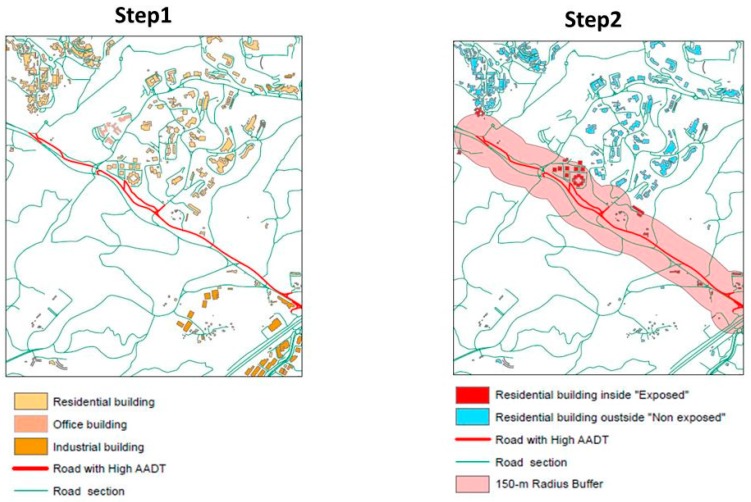
Description of the two successive and complementary steps required to estimate the heavy residential traffic exposure indicator.

Using ArcGis software, the residential traffic exposure indicator for each census block was calculated following the two steps:

(i) First, we modeled the distribution of inhabitants in buildings. The total number of inhabitants living in a given census block was distributed in each residential building located within the census block. To do this, two data sources were used: building volume according to the topographic database (from the National Geographic Institute) and the number of inhabitants per census block according to the national census, collected by the French National Statistics Institute (INSEE). The number of inhabitants per residential building was calculated assuming the equi-distribution to its volume.
(ii) Second, the percentage of inhabitants living within 150 m of a major road was calculated for each census block. More precisely, circles with radii of 150 m were created around each road segment, as shown in Figure 2. The widest radius, of 150 m, was chosen based on studies revealing that NO_2_ concentrations drop off very rapidly within the first 50 m, and are divided by ten after 100 m [37,38]. We intersected these circles (buffers) with the topological database to pinpoint residential buildings located inside each circle which would be considered exposed to high-traffic roads. The total number of exposed inhabitants per census block was obtained by adding together the number of inhabitants of all the residential buildings within the buffers. Finally, the residential traffic exposure indicator was estimated at census block level by dividing the total number of exposed inhabitants by the population of the census block.

**Figure 3:**
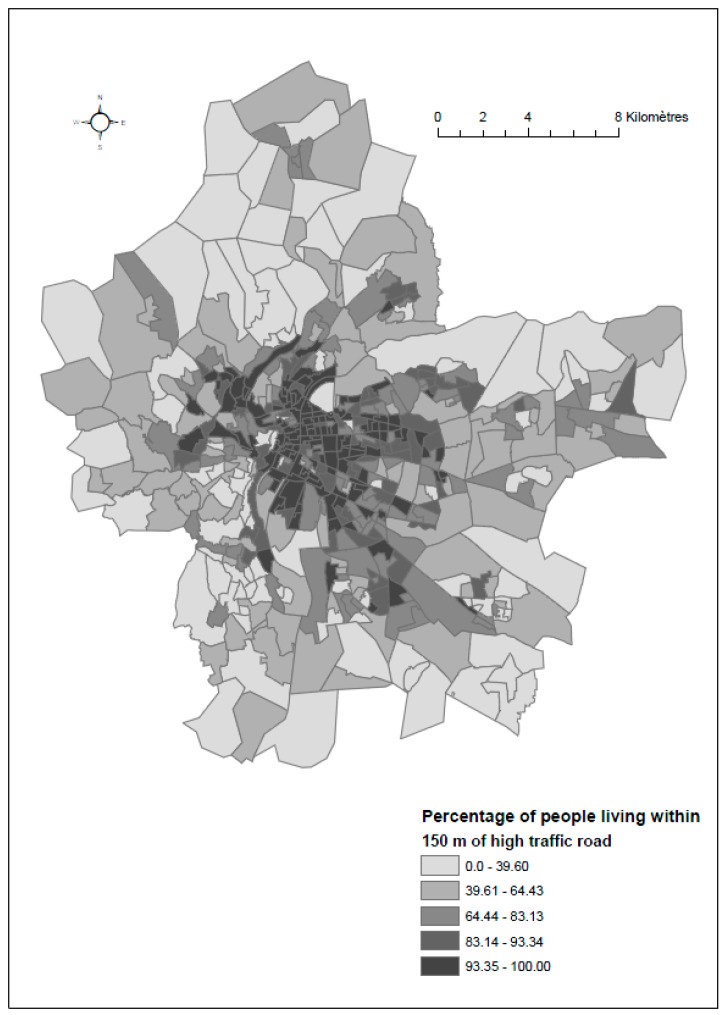
Spatial distribution of the percentage of population living within 150 m of a high traffic road based on quintiles of the distribution.

Figure 3 represents the spatial distribution of the percentage of population exposed to pollution from the high traffic road. 

#### 2.3.3. Noise Exposure

Residential noise exposure includes related noise coming from all sources: road traffic, aircraft traffic, industry and railway. Noise pollution was measured in 2007, in accordance with the European Environmental Noise Directive (END, 2002/49/EC) [39], using GIpSynoise software and various input data including information on road and railway characteristics, topography, meteorological factors and other data on sound reflection and diffraction. Acoustic modeling of noise levels with a spatial resolution of 10 × 10 m at 4 m above ground level, across the Lyon metropolitan area was performed by the Greater Lyon Urban Community (the political body comprising the municipalities of the Lyon metropolitan area). In our study, the metric used to characterize noise in each census block is a population-weighted average noise exposure which combines the European Lden indicator (day-evening-night level, expressed in decibels noted dB) and the estimated population living in each residential building. The Lden indicator corresponds to an assessment of daily exposure over a 24-h period taking into account residents’ increased sensitivity to noise during the evening and night [40]. This noise exposure indicator accommodates census blocks in which fewer people are exposed to higher noise levels, and more people are exposed to lower noise levels. This approach is derived from the definition of total noise load of a population as given in the EEA Technical Report [41]. More details about how these indicators were constructed can be found elsewhere [42]. 

**Figure 4:**
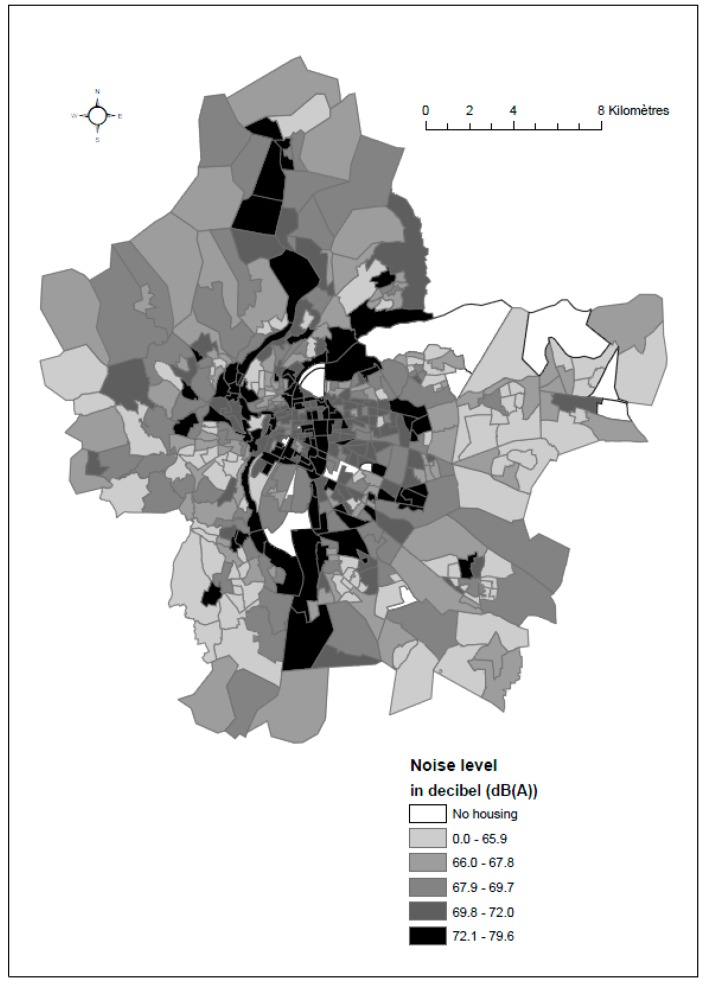
Spatial distribution of noise exposure (the weighted Lden noise indicator) at census block level based on quintiles of the distribution.

Figure 4 presents the spatial distribution of noise exposure and reveals that census blocks with high noise levels (>71 dB(A)) are concentrated in the central and eastern parts of the metropolitan area, whereas census blocks with the lowest levels (<71 dB(A)) are found in the suburbs of the city of Lyon.

#### 2.3.4. Green Spaces

**Figure 5:**
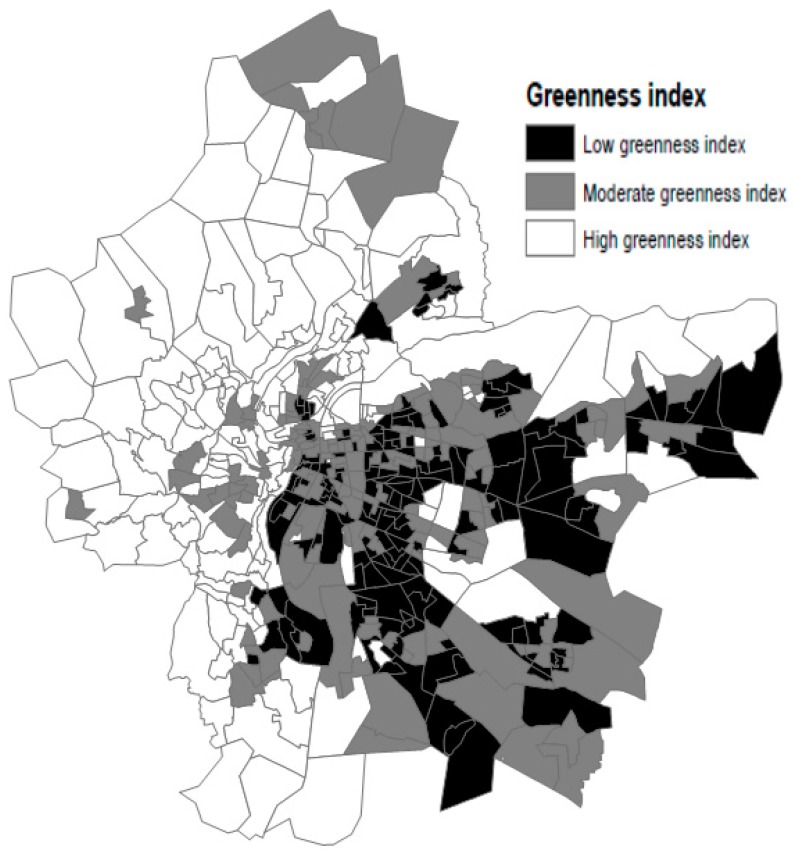
Spatial distribution of green space exposure, at census block level, based on tertiles of the distribution.

Spatial land cover datasets for the Lyon Metropolitan area were processed using ArcMap GIS software (Redlands, CA, USA) in order to produce a green space index (Figure 5). In our study, all the different types of natural areas (e.g., parks, forests) were included. 

More precisely, our greenness index was the percentage of green spaces (km^2^) in the total area of the census block. (More methodological details in [43]).

#### 2.3.5. Socioeconomic Deprivation Index

**Figure 6:**
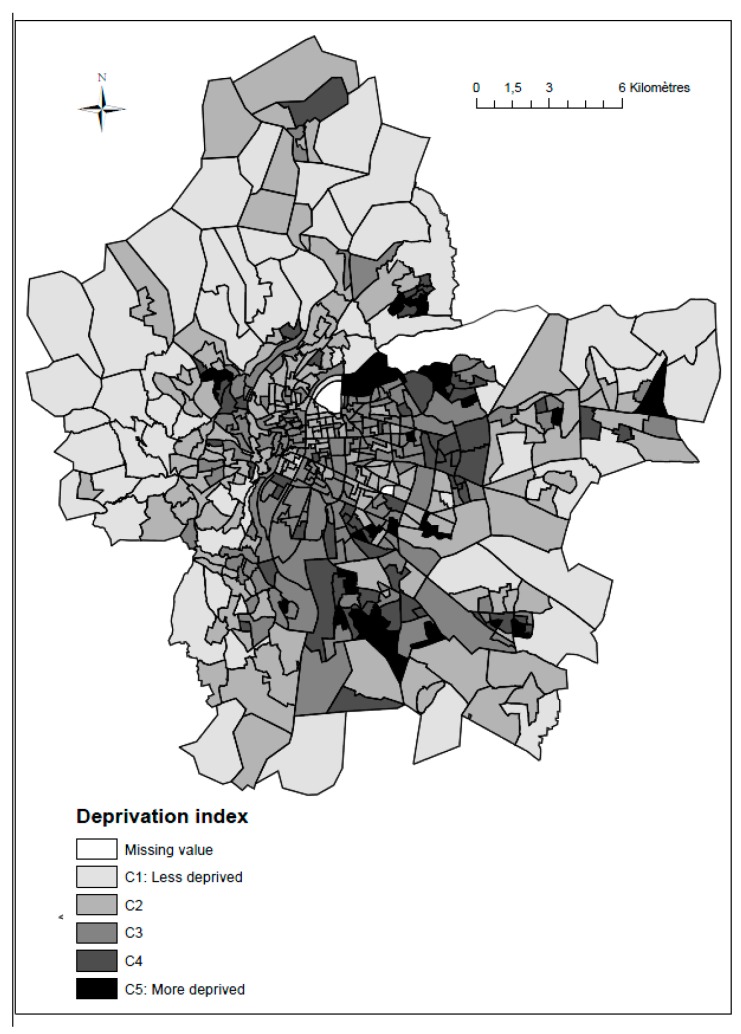
Spatial distribution of the deprivation index, at census block level, based on quintiles of the distribution.

A deprivation index was used to capture the different socioeconomic aspects by combining variables (family structure, household type, immigration status, employment, income, education and housing) collected by the INSEE institute (2006). Principal component analyses were run to combine the socioeconomic variables into an index (More methodological details in [44]). The deprivation index was divided into five categories of census blocks based on quintiles of the distribution. The spatial distribution of the socioeconomic index (Figure 6) reveals a clear gradient from the south-west, the least deprived, to the north-east, the most deprived. 

### 2.4. Statistical Analysis

In order to understand how individual characteristics combined with the neighborhood characteristics measured at a fine spatial scale, may or may not alter the relationship between the objective and the subjective measures of air pollution, a data mining approach was applied.

Two multidimensional methods successively were applied to identify different profiles among our study population. A multiple correspondence analysis (MCA) was performed which is a well-suited technique to analyze qualitative variables only. Table 1 and Table 2 give the list of variables collected via the questionnaire included in the MCA. In addition, the neighborhood variables have been also considered in the MCA as supplementary variables to characterize the place where people live; namely NO_2_, noise, traffic, green space and socioeconomic deprivation.

Following MCA, a Hierarchical Clustering (HC) was applied to create meaningful individual’s profiles step by step. HC is an unsupervised method of clustering that builds a hierarchy of clusters, frequently used after MCA. At each step, the two groups of individuals which are the closest according to the ward distance between categories are merged. This algorithm aims to obtain homogenous individual’s profiles by minimizing and maximizing within and between variabilities, respectively. The final choice of the number of individual’s profiles is also based on the dendrogram.

Preliminary MCA revealed the expected demographic structure in the data. Three groups of respondents were clearly identified: younger people (ages 19–29), middle aged people (ages 30–59) and older people (ages 60 and older). Then, MCA and HC were conducted separately on these 3 groups. Each category resulting from the HC was characterized by the statistically significant supplementary variables Analyses were performed with R software (R-version 3.2.2). The MCA and HC were performed using the ‘factoMineR’ package for R.

Bi-variate analysis realized in our study included two different statistical tests: the mean-comparison test and the non-parametric test for trend across ordered groups; the level of significance was α = 5%. 

## 3. Results

### 3.1. Characteristics of the Study Population

Table 1 provides a description of the main individual characteristics of the population. In all, 968 individuals were considered in the analysis. Thirty-two individuals were excluded due to the high number of missing values; this represents only 3.2% of the total. About 56% of the respondents were women. A quarter of the respondents were between 44 and 59 years old. More than half of the respondents live with a partner (with or without children). About 38% have a low level of education whereas 30% have a high level. About 1/3 are retired and 1/5 are unemployed. Concerning the occupational status, more than 40% of the respondents work (or worked) in their residential municipalities. The means of travel to work is predominantly by car (51%). For 55.8% of the respondents, the commute time ranges from 11 to 30 min, on average. As regards housing characteristics, 3/4 of the respondents live in a house. The year of construction of the dwelling is earlier than 1987 for 58% of the respondents. About 78% of the respondents have resided in the Lyon metropolitan area for at least 10 years. The most frequent reasons why they live in the Lyon metropolitan area are professional reasons (32%), birthplace, meaning that they have always lived in the Lyon metropolitan area (30%) and personal reasons (29%). Regarding health, 27% of respondents reported respiratory problems and 9% reported cardiovascular problems. 

Table 2 summarizes the descriptive statistics of variables related to perception and knowledge about the level of air quality. About 47% of the respondents somewhat agreed with the statement “*the quality of the air you breathe within your residential census block is good*” whereas 24% reported that they did not agree. More than 1/4 reported having knowledge about air quality. Only 6% of respondents did not appreciate the place where they live, while 47% were totally satisfied and 46% were somewhat satisfied. 

### 3.2. Relationship between Subjective and Objective Measures of Air Pollution

**Figure 7:**
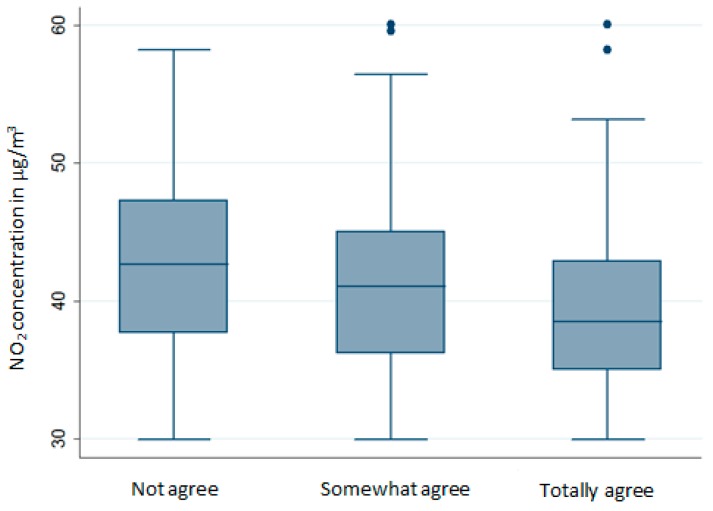
Distribution of NO_2_ concentrations according to the level of satisfaction with air quality at census block level.

The mean NO_2_ concentration estimated at census block level is 40.5 μg/m^3^ (standard deviation = 5.8 μg/m^3^). Statistical analysis reveals a significant increase in NO_2_ concentrations estimated at the census block level with the level of dissatisfaction with air quality reported by the respondents (*p*-value < 0.0001). As shown on the Figure 7, respondents reporting to be satisfied compared to those dissatisfied live in a place with average NO_2_ concentrations equal to 39.3 μg/m^3^ (standard deviation = 5.8 μg/m^3^) and 42.4 μg/m^3^ (standard deviation = 5.5 μg/m^3^), respectively. 

This finding was consistent with the other indicators of environmental pollution, such as the percentage of population living within 150 m of heavy traffic roads. More precisely, the percentage of population exposed to road traffic increased with the level of dissatisfaction of the respondents: the median value was 84.8% vs. 98.7% (*p*-value < 0.0001). Conversely, there is no significant association between the annual average NO_2_ concentrations and the knowledge of the respondents regarding interventions aimed at improving air quality in Lyon Metropolitan area (*p*-value = 0.15).

In addition, respondents living in a census block with a higher density of green spaces were significantly more satisfied with their place of residence than those living in a census block with a lower density (the median value of the green space indicator was 28,996.7 km^2^ vs. 20,759.1 km^2^, respectively; *p*-value < 0.023). Finally, there is a statistically significant association between the level of socioeconomic deprivation and the satisfaction level of their place of residence: respondents living in the more deprived census blocks are less satisfied than those living in the less deprived census blocks (*p*-value < 0.0001). 

### 3.3. Relationship between Subjective and Objective Measures of Air Quality Based on the Demographic Characteristics of Respondents

The multiple correspondence analyses followed by the hierarchical analysis were applied separately among three different age groups: the youngest (ages 18 to 29), the middle age (ages 30 to 59) and the oldest (ages 60 and older): four homogeneous profiles were identified among the youngest group; 4 profiles among the middle aged group and 5 among the oldest group. Detailed descriptions of individual characteristics by profile are given in supplementary Tables S1–S3. Supplementary Figures S1–S3 represent the MCA factor map in two-dimensions (Dim1 × Dim2) among the young, middle and old population, respectively.

Table 3 includes descriptive statistics on knowledge about interventions aimed at improving air quality in Lyon Metropolitan Area, the willingness to move to another place because of poor air quality and the global level of satisfaction with the place where the respondents live.

Among the youngest population, we found consistent results between subjective and objective measures except for in profile 4. Profile 1 includes satisfied individuals as regards air pollution who live in census blocks with NO_2_ concentrations significantly lower than the average; this also corresponds to less deprived census blocks. Profile 2 includes a majority of dissatisfied individuals living in more polluted and more deprived census blocks, though the results are less clear. Individuals in profile 3 reported different satisfaction levels with air quality. However, at the same time, the NO_2_ concentrations of the census blocks where they live are also different. We found a significant increase of air pollution with the satisfaction level (*p*-value < 0.015). In addition, for these 3 profiles, the results are also consistent with other contextual variables: when NO_2_ concentrations are high, the percentage of people living close to a highway is also high, and the green space indicator is low (data not shown). These first 3 profiles are defined with various combinations of individual characteristics (gender, place of work, means of travel to work, etc.—see additional Table 1 for more details). Profile 4 is the only one that presents no clear relationship between air quality satisfaction and measures or air pollution: the individuals are somewhat satisfied with air quality whereas the NO_2_ concentrations vary between [36.4, 45.5] μg/m^3^ based on the interquartile interval of NO_2_ concentrations. This corresponds to jobseekers with a low level of education, living with a partner and with children. 

Among the middle aged population (age 30 to 59), as with the youngest population, consistent results between satisfaction and measures of air quality were found for individuals in profile 2 and profile 4, and to a lesser extent, in profile 1 (borderline significant *p*-value = 0.08). Profile 4 (like profile 3 among the youngest), revealed a significant increase in NO_2_ concentrations with the level of air quality satisfaction (*p*-value = 0.031)—most of the census blocks are less deprived. Profile 2 includes individuals somewhat or not at all satisfied with the air quality and who live in the more exposed census blocks. The relationship is less clear for individuals in profile 3: the lowest level of NO_2_ concentrations was measured among those partially satisfied with the air quality. More precisely, this corresponds to employed individuals (skilled manual or non-manual employees) with a low level of education, who work at home or in a municipality of the Rhone-Ales department (but not the municipality of residence).

Among the oldest population, there is globally no significant difference between the NO_2_ concentrations and the level of satisfaction; this result is confirmed in profile 1 and profile 2. Profile 1 includes employed individuals who have lived in Lyon for more than 10 years and who do not consider moving to another place, working in a municipality of the Rhône-Alpes department (but not the municipality of residence). In profile 2, people are retired with a low level of education, reporting no respiratory or cardiovascular health problems. Although the results are not statistically significant, group 3 includes individuals living in census blocks globally less exposed to NO_2_ and with a lower level of socioeconomic deprivation; the NO_2_ concentrations were lower for those who were totally satisfied with the air quality. Finally, of the 138 individuals in profiles 4 and 5, only 2 people reported being totally satisfied with the air quality, whereas the 7 individuals who said they were somewhat satisfied with the air quality live in census blocks with a low level of NO_2_ concentrations but a high level of socioeconomic deprivation. In profiles 4 and 5, individuals have a low level of education and report more frequent respiratory health problems. The two profiles also have specific characteristics: profile 4 included single women with no children whereas in profile 5, the individuals reported cardiovascular health problems in addition to respiratory health problems.

## 4. Discussion

The main goal of this study was to investigate the relationship between the subjective and objective measures of air pollution, and to explore the existence or absence of this relationship within homogenous populations in term of individual characteristics. 

The key finding of our study is that there is a correlation between subjective measures of air quality (the level of satisfaction with air quality) and the objective measures of air quality (average concentrations of NO_2_ modeled at the residential census block level). In other words, in many population sub-groups with different combinations of individual characteristics, we found a significant relationship between subjective and objective measures of air pollution. The relationship is less clear among the oldest population, which confirms previous findings. 

Several studies have investigated whether there is an association between perceived air quality reported and individual characteristics, including age. A recent study [33] did not reveal any significant association between age and perceived air quality, while in another study the authors found that people aged 20 to 34 had poorer perceived air quality compared to the oldest population [19]. In our study, the people’s age constitutes one of the determinants of perception but cannot be interpreted independently of other individual factors. Among the youngest and the middle aged populations, the level of education was identified as a significant individual characteristic. More particularly, individuals included in the profiles showing a discrepancy between measures and perception have a low level of education. In the literature investigating the air pollution effect, socioeconomic indicators are recognized to be important factors in studies dealing with perceived air quality because these factors may affect the susceptibility and/or the exposure to environmental pollution [45]. More precisely, in the literature the effect of education level on perceived air quality has been largely addressed and the results remain contrasted. Previous studies have reported higher levels of education to be associated with higher annoyance levels or poorer perceived air quality [19,30] and conversely, others have demonstrated that a low level of education was a significant determinant of annoyance with perceived air pollution [46,47]. More recently, Guo et al. [48] demonstrated that parents with a high level of education are more likely to be less satisfied with air quality; the authors argued that level of education could influence the economic, vocational and social status. In 2009, Badland et al. [49] explained that people with a high level of education are more likely to be concerned by environmental issues.

Among the oldest population, a low level of education also appears to be a characteristic of profiles that revealed no significant association between perceived air quality and air pollution measures. In addition to a low level of education, we found that respiratory health problems were a significant characteristic of two profiles among the oldest population: they reported suffering from respiratory health problems more frequently than the average. Other specific characteristics include unemployment (profile 4 among the youngest population), occupational status (skilled manual or non-manual employees in profile 3 among the middle aged population), single women with no children (profile 4 among the oldest population) and cardiovascular health problems (profile 5 among the oldest population); these variables characterize the profiles for which there is no consistent correlation between perceived air quality and the measures of air pollution. Previous studies reported a significant association between perceived air quality and self-reported health status [50,51]: perceived health risk related to air pollution was found to be associated with the level of education and occupational status. Regarding gender, some authors have argued that women, in particular those with young children, tend to be more aware of, or more anxious about, environmental risks, and suggest that women are more environmentally aware than men [28]. Other authors have suggested that women tend to have a better sense of smell than men [52]. 

There are several limitations to our study. First, the homogenous profiles were drawn up and analyzed using individual data collected from a telephone survey, which inevitably introduced a selection bias in the study population. Second, the air pollution, modeled at the residential census block level, does not take into account the daily mobility of the population, meaning that we assigned the same NO_2_ concentration values to the individuals living in the same census blocks. The exposure misclassification will be lower for individuals who work close to their homes. However, our measures of NO_2_ concentrations based on dispersion models gave average concentrations at the census block level; the spatial disparities of the Lyon metropolitan area were taken into account when investigating the relationship between individual satisfaction with air quality and air pollution measures modeled at the residential census block level. Third, we used a basic opinion question on air quality to assess perceived air quality, while several studies reported no significant association with the objective measure [29,53]. Finally, one limitation of our study deals with the statistical power; even if several significant results were found, we cannot rule out the possibility that other results would have been significant with a larger sample size.

## 5. Conclusions

This study revealed the combination of individual characteristics identifying profiles of respondents within which the level of air quality satisfaction and the NO_2_ concentrations measured are consistent. We also identified that age combined with a low level of education and unemployment, or women or health problems as well as the neighborhood deprivation index influence the level of air quality satisfaction. This will help develop targeted risk communication messages. It also shows that objective and subjective measures of pollution are complementary indicators to fully assess the health effects of air pollution. It confirms the importance of subjective measures of air pollution for future research and that an opinion question is sufficient to obtain an overall view of the level of air quality satisfaction.

## Figures and Tables

**Table 1:** Descriptive statistics of socio-demographic characteristics of the study population.

Characteristics	Frequency	%
**Gender**		
Men	440	44.0
Women	560	56.0
**Age (years)**		
18–29	147	14.7
30–44	220	22.0
45–59	251	25.1
60–74	229	22.9
>74	153	15.3
**Family status**		
Live with a partner without children	309	30.9
Live with a partner with children	287	28.7
Live alone without children	257	25.7
Live with parent	84	8.4
Single parent families	56	5.6
Live with a roommate	7	0.7
**Level of education**		
Low level	384	38.4
Intermediate level	321	32.1
High level	295	29.5
**Occupational status**		
Retired	339	33.9
Student	61	6.1
Employed	460	46.0
Unemployed-inactive	140	14.0
**Housing (1 missing value)**		
House	258	25.8
Apartment	739	74.0
Other type of housing	2	0.2
**Heating mode (4 missing values)**		
Wood	37	3.7
Fuel oil	76	7.6
Electric	251	25.2
Gas	592	59.4
Other	40	4.0
**Number of years that they have lived in the metropolitan area (4 missing values)**		
<1 year	8	0.8
1–2 years	14	1.4
2–4 years	77	7.7
5–9 years	121	12.1
≥10 years	776	77.9
**Respiratory health problem (3 missing values)**		
no	731	73.3
yes	263	26.4
Do not know	3	0.3
**Cardiovascular health problem**		
no	912	91.2
yes	88	8.8
**Occupation**		
Tradespeople-Shopkeepers	26	5.7
Managers	108	23.5
Employees	137	29.8
Manual workers	64	13.9
Non-manual workers	125	27.2
**Work location (1 missing value)**		
At home	36	7.8
In the municipality of residence	187	48.6
In a municipality of the Rhône-Alpes department	192	90.4
In a municipality not in the Rhône-Alpes department	12	2.6
Other place of work	32	7.0
**Means of travel to work (37 missing values)**		
Public transportation (Bus, tramway, metro)	91	21.5
Car	230	54.4
Bicycle	31	7.3
Motorbike	7	1.7
By foot	53	12.5
Other	11	2.6

**Table 2:** Descriptive statistics of variables dealing with perception and satisfaction.

Questions	Answers	Frequency	%
Would you say that the quality of the air you breathe within your residential census block is good (3 missing values)	Yes totally agree	228	22.9
	Somewhat agree	471	47.2
	Do not agree	244	24.5
	No opinion—do not know	54	5.4
Would you ever consider living in another place where air quality is better (7 missing values)	Yes	289	29.1
	No	673	67.8
	No opinion—do not know	31	3.1
Are you aware of actions or interventions aimed at improving air quality in the Lyon MA (3 missing values)	Yes	267	26.8
	No	729	73.2
Would you say that you are satisfied with the place where you live	Yes totally agree	472	47.2
	Somewhat agree	460	46.0
	Do not agree	61	6.1
	No opinion—do not know	7	0.7

**Table 3:** (**a**) Descriptive statistics of perception, satisfaction and knowledge about air quality by the profile among the youngest (ages 18 to 29 years); (**b**) Descriptive statistics of perception, satisfaction and knowledge about air quality by the profile among the middle age (ages 30 to 59 years); (**c**) Descriptive statistics of perception, satisfaction and knowledge about air quality by the profile among the oldest (≥60 years).

**(a)**						
			**Profile 1** **(*n* = 31)**	**Profile 2** **(*n* = 43)**	**Profile 3** **(*n* = 48)**	**Profile 4** **(*n* = 24)**
Perceived Air quality		Totally agree	67.74	4.65	20.83	12.50
		Somewhat agree	16.13	41.86	56.25	66.67
		Not agree	6.45	51.16	22.92	16.67
		Do not know	9.68	2.33	0.00	4.17
Satisfaction with the residential place		Totally agree	77.42	23.26	43.75	41.67
		Somewhat agree	16.13	60.47	56.25	58.33
		Not agree	0.00	16.28	0.00	0.00
		Do not know	6.45	0.00	0.00	0.00
Living in another place		yes	16.13	81.40	22.92	25.00
		no	83.87	18.60	64.58	66.67
		Do not know	0.00	0.00	12.50	8.33
Knowledge about air quality		yes	35.48	23.26	27.08	29.17
		no	64.52	76.74	72.92	70.83
**(b)**						
			**Profile 1** **(*n* = 121)**	**Profile 2** **(*n* = 124)**	**Profile 3** **(*n* = 121)**	**Profile 4** **(*n* = 102)**
Perceived Air quality		Totally agree	28.10	0.00	33.06	15.69
		Somewhat agree	52.07	44.35	47.93	44.12
		Not agree	16.53	54.03	10.74	34.31
		Do not know	3.31	1.61	8.26	5.88
Satisfaction with the residential place		Totally agree	50.41	12.90	57.02	39.22
		Somewhat agree	48.76	70.16	38.84	51.96
		Not agree	0.83	16.94	4.13	8.82
		Do not know	0.00	0.00	0.00	0.00
Living in another place		yes	28.93	65.32	20.66	42.16
		no	68.60	29.84	76.03	52.94
		Do not know	2.48	4.84	3.31	4.90
Knowledge about air quality		yes	49.59	33.06	26.45	23.53
		no	50.41	66.94	73.55	76.47
**(c)**						
		**Profile 1** **(*n* = 34)**	**Profile 2** **(*n* = 99)**	**Profile 3** **(*n* = 84)**	**Profile 4** **(*n* = 103)**	**Profile 5** **(*n* = 35)**
Perceived Air quality	Totally agree	29.41	59.60	23.81	1.94	0.00
	Somewhat agree	41.18	27.27	59.52	73.79	20.00
	Not agree	29.41	6.06	11.90	11.65	80.00
	Do not know	0.00	7.07	4.76	12.62	0.00
Satisfaction with the residential place	Totally agree	38.24	268.57	44.05	52.43	17.14
	Somewhat agree	55.88	11.43	54.76	47.57	40.00
	Not agree	5.88	0.00	1.19	0.00	37.14
	Do not know	0.00	2.86	0.00	0.00	5.71
Living in another place	yes	17.65	1.01	14.29	4.85	57.14
	no	79.41	98.99	85.71	92.23	40.00
	Do not know	2.94	0.00	0.00	2.91	2.86
Knowledge about air quality	yes	23.53	5.15	44.05	11.65	8.57
	no	76.47	94.85	55.95	88.35	91.43

## Supplementary Materials

The following are available online at www.mdpi.com/1660-4601/14/12/1559/s1, Figure S1: MCA factor map in two-dimensions (Dim1 × Dim2) among the young population, Figure S2: MCA factor map in two-dimensions (Dim1 × Dim2) among the middle-age population, Figure S3: MCA factor map in two-dimensions (Dim1 × Dim2) among the older population, Table S1: Description of the profile 1–4 among the youngest (ages 18 to 29), Table S2: Description of the profile 1–4 among the middle age (ages 30 to 59), Table S3: Description of the profile 1–5 among the oldest (≥60 years).
