# Supplementary material for: Do Individual and Neighborhood Characteristics Influence Perceived Air Quality?
Source: Int J Environ Res Public Health. 2017 Dec 12;14(12):1559. doi: 10.3390/ijerph14121559 (PMC5750977; doi:10.3390/ijerph14121559)
Supplement: Supplementary file 1 [file ijerph-14-01559-s001.pdf]

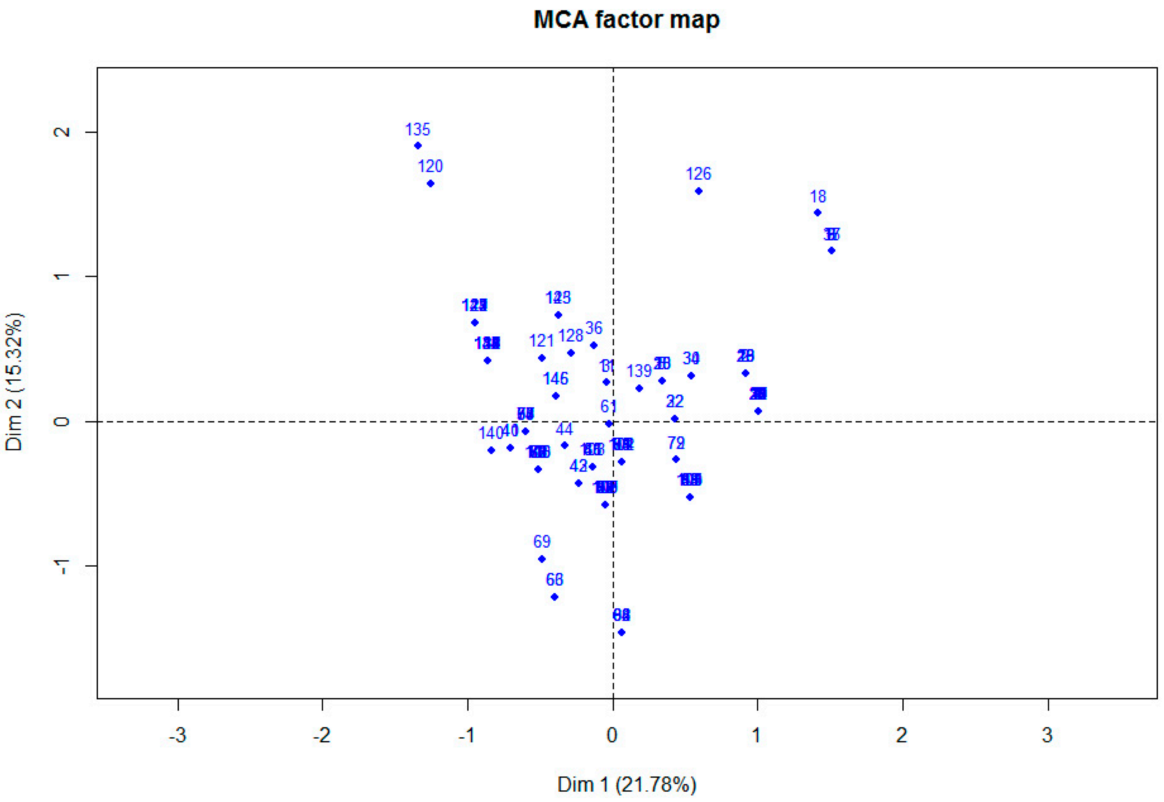

Figure S1: MCA factor map in two-dimensions (Dim1 x Dim2) among the young population.

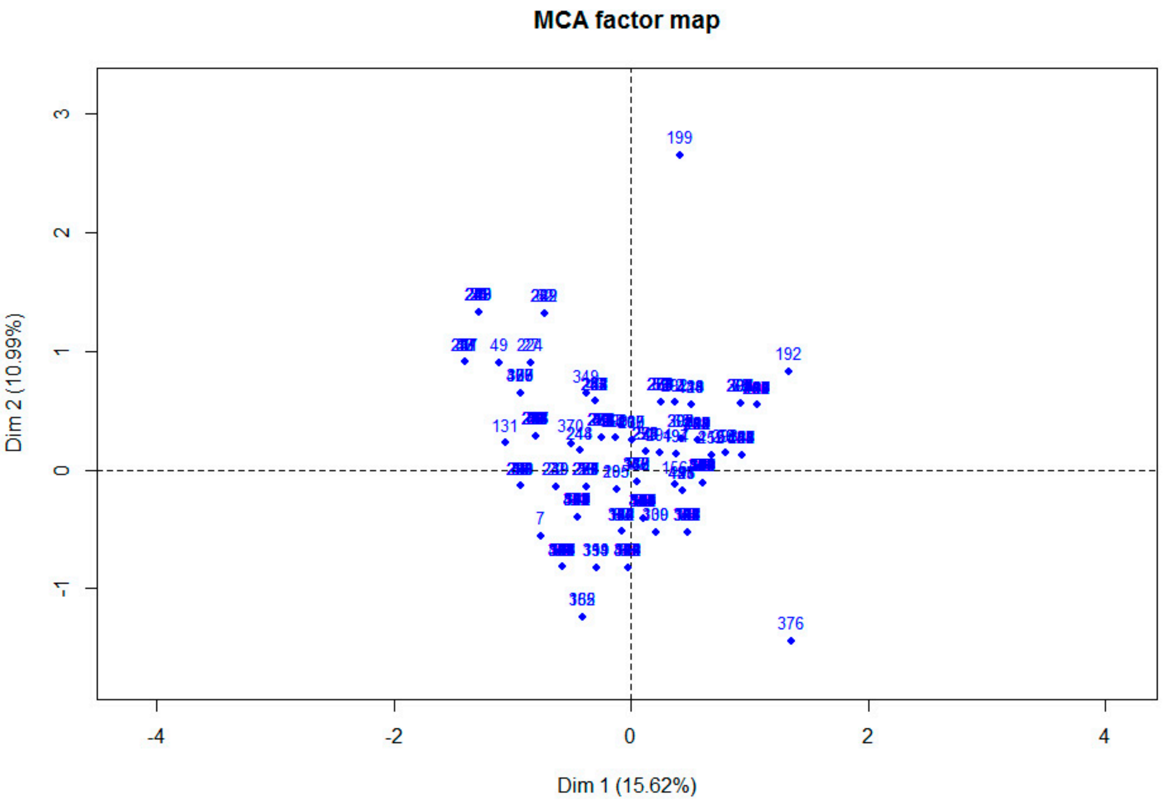

Figure S2: MCA factor map in two-dimensions (Dim1 x Dim2) among the middle-age population.

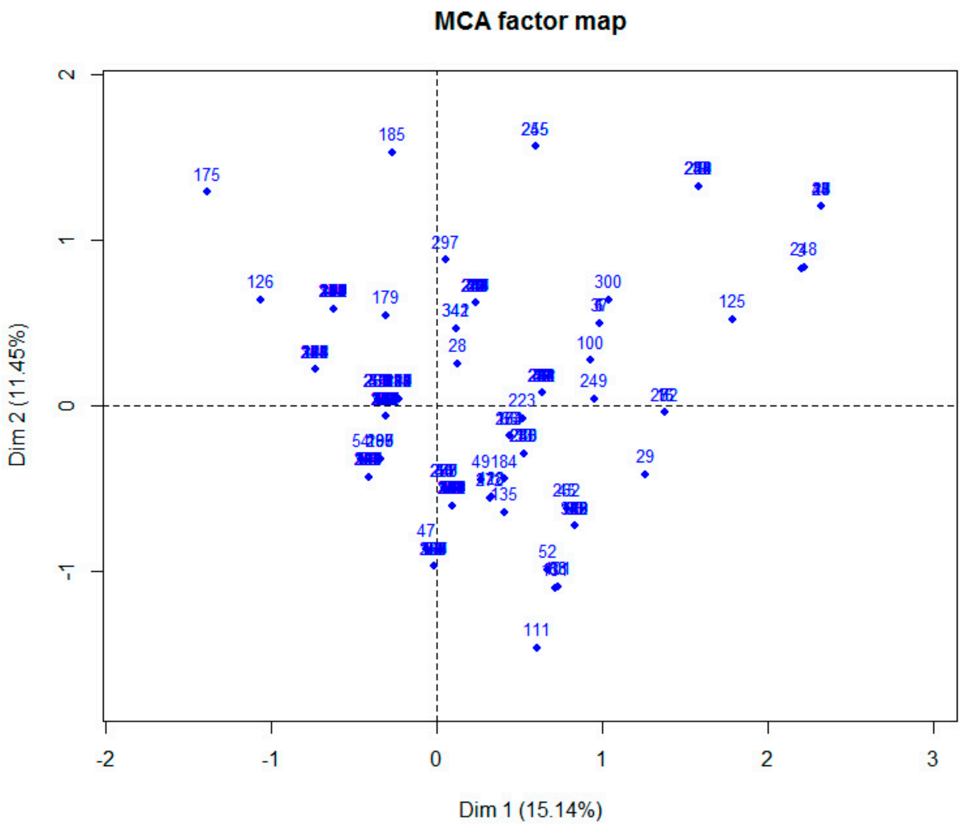

Figure S3: MCA factor map in two-dimensions (Dim1 x Dim2) among the older population.

Table S1a: Description of the **profile 1** among the youngest (18–29 years)

**Variable**: *modality*	Cla/Mod [ɸ]	Mod/Cla [¥]	Global [§]	*p* value
**Travel mean**: *Car*	55.81	77.42	29.45	<0.00001
**Perceived Air quality**: *Totally agree*	58.33	67.74	24.66	<0.00001
**Housing**: *House*	57.14	51.61	19.18	<0.00001
**Satisfaction with the residential place**: *Totally agree*	36.92	77.42	44.52	<0.0001
**Living in another place**: *No*	32.10	83.87	55.48	<0.001
**Gender**: *Man*	32.43	77.42	50.68	<0.001
**Work location**: *In a municipality not in the Rhône-Alpes department*	39.53	54.84	29.45	<0.001
**Work location**: *Other place of work*	100.00	12.90	2.74	<0.01
**Occupational status**: *Employed*	32.81	67.74	43.84	<0.01
**Number years living in Lyon**: *>10 years*	27.78	80.65	61.64	<0.05
**Respiratory health problem**: *No*	25.47	87.10	72.60	<0.05
**Level of Education**: *Baccalaureat*	0.00	0.00	10.27	<0.05
**Heating mode**: *Electric*	10.20	16.13	33.56	<0.05
**Respiratory health problem**: *Yes*	7.69	9.68	26.71	<0.05
**Number years living in Lyon**: *5–9 years*	3.85	3.23	17.81	<0.05
**Work location**: *In the municipality of residence*	11.27	25.81	48.63	<0.01
**Living in another place**: *Yes*	8.77	16.13	39.04	<0.01

**Perceived Air quality**: *Not agree*	5.13	6.45	26.71	<0.01

Table S1b: Description of the **profile 2** among the youngest (18–29 years)

**Variable**: *modality*	Cla/Mod ɸ	Mod/Cla ¥	Global §	*p* value
**Living in another place:** *Yes*	61.40	81.40	39.04	<0.00001
**Occupational status:** *Employed*	56.25	83.72	43.84	<0.00001
**Number years living in Lyon:** *5–9 years*	76.92	46.51	17.81	<0.00001
**Housing:** *Apartment*	36.44	100.00	80.82	<0.0001
**Travel mean:** *Bicycle*	84.62	25.58	8.90	<0.0001
**Perceived Air quality:** *not agree*	56.41	51.16	26.71	<0.0001
**Family status:** *Live with partner without children*	65.22	34.88	15.75	<0.001
**Satisfaction with the residential place:** *Not agree*	100.00	16.28	4.79	<0.001
**Level of education:** *High*	61.11	25.58	12.33	<0.01
**Family status:** *Live alone without children*	57.89	25.58	13.01	<0.01
**Occupation:** *Tradespeople-Shopkeepers*	100.00	9.30	2.74	<0.01
**Occupation:** *Employees*	50.00	34.88	20.55	<0.01
**Level of education:** *Low*	6.25	2.33	10.96	<0.05
**Occupational status:** *Unemployed*	0.00	0.00	8.22	<0.05
**Level of education:** *Baccalaureat*	0.00	0.00	8.22	<0.05

**Satisfaction with the residential place:** *Totally agree*	15.38	23.26	44.52	<0.001
**Occupational status:** *Student*	12.07	16.28	39.73	<0.001
**Perceived Air quality:** *Totally agree*	5.56	4.65	24.66	<0.001

Table S1c: Description of the **profile 3** among the youngest (18–29 years)

**Variable**: *modality*	Cla/Mod [ϕ]	Mod/Cla [¥]	Global [§]	*p* value
**Occupational status:** *Student*	70.69	85.42	39.73	<0.00001
**Family status:** *With parent*	56.96	93.75	54.11	<0.00001
**Occupation:** *Missing value*	59.42	85.42	47.26	<0.00001
**Travel mean:** *Public transport*	63.46	68.75	35.62	<0.00001
**Number years living in Lyon :** *>10 years*	46.67	87.50	61.64	<0.00001
**Work location:** *In the municipality of residence*	50.70	75.00	48.63	<0.00001
**Level of education:** *Low*	75.00	25.00	10.96	<0.001
**Level of education:** *Baccalaureat*	73.33	22.92	10.27	<0.001
**Respiratory health problem:** *Yes*	48.72	39.58	26.71	<0.05
**Travel mean:** *Bicycle*	7.69	2.08	8.90	<0.05
**Level of education:** *High*	11.11	4.17	12.33	<0.05
**Occupation:** *Employee*	16.67	10.42	20.55	<0.05

**Respiratory health problem:** *No*	27.36	60.42	72.60	<0.05
**Occupation:** *Manual worker*	6.67	2.08	10.27	<0.05
**Occupation:** *Manager*	0.00	0.00	7.53	<0.05
**Occupation:** *Non-manual worker*	5.88	2.08	11.64	<0.01
**Occupational status:** *unemployed*	0.00	0.00	8.22	<0.01
**Travel mean:** *Car*	16.28	14.58	29.45	<0.01
**Living in another place:** *Yes*	19.30	22.92	39.04	<0.01
**Family status:** *Live alone without children*	5.26	2.08	13.01	<0.01
**Family status:** *Live with partner with children*	0.00	0.00	12.33	<0.001
**Number years living in Lyon:** *5–9 years*	3.85	2.08	17.81	<0.001
**Family status:** *Live with partner without children*	0.00	0.00	15.75	<0.0001

Table S1d: Description of the **profile 4** among the youngest (18–29 years)

**Variable**: *modality*	Cla/Mod[ɸ]	Mod/Cla[¥]	Global[§]	*p* value
**Work location**: *Missing value*	100.00	100.00	16.44	<0.00001
**Travel mean**: *Missing value*	96.00	100.00	17.12	<0.00001
**Occupational status**: *Unemployed*	100.00	50.00	8.22	<0.00001
**Occupational status**: *Inactive*	100.00	16.67	2.74	<0.001
**Family status**: *Live with partner with children*	44.44	33.33	12.33	<0.001
**Level of education**: *Baccalaureat*	50.00	25.00	8.22	<0.01
**Level of education**: *Low*	57.14	16.67	4.79	<0.05
**Occupation**: *Manual worker*	40.00	25.00	10.27	<0.05
**Perceived Air quality**: *Somewhat agree*	24.24	66.67	45.21	<0.05
**Family status**: *Live alone without children*	0.00	0.00	13.01	<0.05
**Heating mode**: *Gas*	9.76	33.33	56.16	<0.05
**Travel mean**: Car	0.00	0.00	29.45	<0.00001
**Location of work**: *In a municipality not in the Rhône-Alpes department*	0.00	0.00	29.45	<0.00001
**Travel mean**: *Public transport*	0.00	0.00	35.62	<0.00001
**Occupational status**: *Student*	0.00	0.00	39.73	<0.00001
**Occupational status**: *Employed*	0.00	0.00	43.84	<0.00001

**Location of work**: *In my residential municipality*	0.00	0.00	48.63	<0.00001

Legend: [ϕ] Percentage of individuals with the modality belonging to the profile; [¥] Percentage of individuals of the profile with the modality; [§] Percentage of the modality among the oldest.

Table S2a: Description of the **profile 1** among the middle age (ages 30 to 59)

**Variable**: *modality*	Cla/Mod [ϕ]	Mod/Cla [¥]	Global [§]	*p* value
**Travel mean:** *Car*	50.53	79.34	40.51	<0.00001
**Occupation:** *Manager*	68.42	53.72	20.26	<0.00001
**Occupational status:** *Employed*	33.52	100.00	76.97	<0.00001
**Level of education:** *High*	50.00	48.76	25.16	<0.00001
**Housing:** *House*	47.29	50.41	27.51	<0.00001
**Work location:** *In a municipality not in the Rhône-Alpes department*	41.46	56.20	34.97	<0.00001
**Work location:** *Other place of work*	70.83	14.05	5.12	<0.00001
**Knowledge about air quality:** *Yes*	38.22	49.59	33.48	<0.0001
**Gender:** *Man*	35.50	58.68	42.64	<0.0001
**Level of education:** *Intermediate*	48.78	16.53	8.74	<0.001
**Travel mean:** *Motorbike*	85.71	4.96	1.49	<0.01
**Living in another place:** *No*	31.20	68.60	56.72	<0.01
**Family status:** *Live with partner with children*	31.71	64.46	52.45	<0.01
**Occupation:** *Tradespeople-Shopkeepers*	54.55	9.92	4.69	<0.01

**Satisfaction with the residential place:** *Totally agree*	32.80	50.41	39.66	<0.01
**Perceived Air quality:** *Totally agree*	37.36	28.10	19.40	<0.01
**Occupational status:** *Retired*	0.00	0.00	2.99	<0.05
**Living in another place:** *Yes*	18.92	28.93	39.45	<0.01
**Level of education:** *Baccalaureat*	7.50	2.48	8.53	<0.01
**Family status:** *Live alone without children*	11.43	6.61	14.93	<0.01
**Perceived Air quality:** *Not agree*	14.81	16.53	28.78	<0.001
**Satisfaction with the residential place:** *Not agree*	2.78	0.83	7.68	<0.001
**Occupation:** *Manual worker*	7.94	4.13	13.43	<0.001
**Level of education:** *Low*	10.23	7.44	18.76	<0.0001
**Travel mean:** *Public transport*	1.52	0.83	14.07	<0.00001
**Housing:** *Apartment*	17.40	48.76	72.28	<0.00001
**Occupation:** *Employee*	1.77	1.65	24.09	<0.00001

Table S2b: Description of the **profile 2** among the middle age (ages 30 to 59)

**Variable**: *modality*	Cla/Mod [ɸ]	Mod/Cla [¥]	Global [§]	*p* value
**Work location:** *In the municipality of residence*	63.43	68.55	28.57	<0.00001
**Housing:** *House*	35.40	96.77	72.28	<0.00001
**Occupational status:** *Employed*	33.80	98.39	76.97	<0.00001
**Perceived Air quality:** *Not agree*	49.63	54.03	28.78	<0.00001
**Living in another place:** *Yes*	43.78	65.32	39.45	<0.00001
**Travel mean:** *By foot*	64.10	20.16	8.32	<0.00001
**Occupation:** *Non-manual worker*	45.05	40.32	23.67	<0.00001
**Satisfaction with the residential place:** *Somewhat agree*	35.37	70.16	52.45	<0.00001
**Satisfaction with the residential place:** *Not agree*	58.33	16.94	7.68	<0.0001
**Level of education:** *Intermediate*	43.16	33.06	20.26	<0.0001
**Travel mean:** *Public transport*	46.97	25.00	14.07	<0.001
**Travel mean:** *Bicycle*	65.00	10.48	4.26	<0.001
**Respiratory health problem:** *Yes*	37.19	36.29	25.80	<0.01
**Level of education:** *Baccalaureat*	83.33	4.03	1.28	<0.01
**Occupational status:** *Retired*	0.00	0.00	2.99	<0.05
**Level of education:** *Low*	4.17	0.81	5.12	<0.01
**Respiratory health problem:** *No*	22.67	62.90	73.35	<0.01

**Heating mode:** *Wood*	0.00	0.00	5.12	<0.001
**Work location:** *In a municipality of the Rhône-Alpes department*	15.85	20.97	34.97	<0.001
**Occupational status:** *Unemployed*	0.00	0.00	8.53	<0.00001
**Occupational status:** *Inactive*	0.00	0.00	9.17	<0.00001
**Living in another place:** *No*	13.91	29.84	56.72	<0.00001
**Satisfaction with the residential place:** *Totally agree*	8.60	12.90	39.66	<0.00001
**Perceived Air quality:** *Totally agree*	0.00	0.00	19.40	<0.00001
**Housing:** *House*	3.10	3.23	27.51	<0.00001

Table S2c: Description of the **profile 3** among the middle age (ages 30 to 59)

**Variable**: *modality*	Cla/Mod $^{\phi}$	Mod/Cla $^{¥}$	Global §	*p* value
**Occupation:** *Employee*	61.06	57.02	24.09	<0.00001
**Occupational status:** *Employed*	32.69	97.52	76.97	<0.00001
**Work location:** *In the municipality of residence*	42.68	57.85	34.97	<0.00001
**Work location:** *At home*	68.75	18.18	6.82	<0.00001
**Living in another place:** *No*	34.59	76.03	56.72	<0.00001
**Level of education:** *Low*	47.73	34.71	18.76	<0.00001
**Occupation:** *Manual worker*	52.38	27.27	13.43	<0.00001

**Travel mean:** *Public transport*	51.52	28.10	14.07	<0.00001
**Level of education:** *Baccalaureat*	60.00	19.83	8.53	<0.00001
**Satisfaction with the residential place:** *Totally agree*	37.10	57.02	39.66	<0.00001
**Perceived Air quality:** *Totally agree*	43.96	33.06	19.40	<0.0001
**Heating mode:** *Other*	70.59	9.92	3.62	<0.001
**Respiratory health problem:** *No*	29.36	83.47	73.35	<0.01
**Work location:** *Other place of work*	4.17	0.83	5.12	<0.01
**Respiratory health problem:** *Yes*	15.70	15.70	25.80	<0.01
**Level of education:** *Intermediate*	12.63	9.92	20.26	<0.001
**Occupational status:** *Unemployed*	5.00	1.65	8.53	<0.001
**Satisfaction with the residential place:** *Somewhat agree*	19.11	38.84	52.45	<0.001
**Occupation: non manual worker**	12.61	11.57	23.67	<0.001
**Occupational status:** *Inactive*	0.00	0.00	9.17	<0.00001
**Living in another place:** *Yes*	13.51	20.66	39.45	<0.00001
**Perceived Air quality:** *Not agree*	9.63	10.74	28.78	<0.00001
**Level of education:** *High*	7.63	7.44	25.16	<0.00001
**Occupation:** *Manager*	2.11	1.65	20.26	<0.00001

Table S2d: Description of the **profile 4** among the middle age (ages 30 to 59)

**Variable**: *modality*	Cla/Mod ϕ	Mod/Cla ¥	Global §	*p* value
**Work location:** *Missing value*	96.23	100.00	22.60	<0.00001
**Travel mean:** *Missing value*	74.45	100.00	29.21	<0.00001
**Occupation:** *Missing value*	93.85	59.80	13.86	<0.00001
**Occupational status:** *Inactive*	100.00	42.16	9.17	<0.00001
**Occupational status:** *Unemployed*	95.00	37.25	8.53	<0.00001
**Occupational status:** *Retired*	100.00	13.73	2.99	<0.00001
**Level of education:** *Low*	54.17	12.75	5.12	<0.001
**Knowledge about air quality:** *Yes*	15.29	23.53	33.48	<0.05
**Travel mean:** *Bicycle*	0.00	0.00	4.26	<0.01
**Work location:** *Other place of work*	0.00	0.00	5.12	<0.01
**Work location**: *At home*	0.00	0.00	6.82	<0.001
**Occupation:** *Non-manual worker*	9.01	9.80	23.67	<0.0001
**Travel mean:** *By foot*	0.00	0.00	8.32	<0.0001
**Occupation:** *Manager*	7.37	6.86	20.26	<0.0001
**Occupation:** *Employee*	6.19	6.86	24.09	<0.00001
**Travel mean:** *Public transport*	0.00	0.00	14.07	<0.00001

**Work location:** *In the municipality of residence*	0.00	0.00	28.57	<0.00001
**Work location:** *In a municipality of the Rhône-Alpes department*	0.00	0.00	34.97	<0.00001
**Travel mean:** *Car*	0.00	0.00	40.51	<0.00001
**Occupational status:** *Employed*	0.00	0.00	76.97	<0.00001

Legend: ϕ Percentage of individuals with the modality belonging to the profile; ¥ Percentage of individuals of the profile with the modality; § Percentage of the modality among the oldest.

Table S3a: Description of the **profile 1** among the oldest (≥60 years)

**Variable**: *modality*	Cla/Mod ϕ	Mod/Cla ¥	Global §	*p* value
**Work location:** *Missing value*	100.00	100.00	91.05	<0.00001
**Occupation:** *Missing value*	100.00	99.71	90.79	<0.00001
**Travel mean:** *Missing value*	99.14	100.00	91.84	<0.00001
**Occupational status:** *Retired*	100.00	93.35	85.00	<0.00001
**Level of education:** *Low*	98.39	17.63	16.32	<0.05
**Perceived Air quality:** *Totally agree*	94.06	59.54	57.63	<0.05
**Perceived Air quality:** *Somewhat agree*	86.33	34.68	36.58	<0.05
**Work location:** *At home*	0.00	0.00	0.79	<0.001
**Occupation:** *Tradespeople - Shopkeepers*	0.00	0.00	0.79	<0.001
**Work location: other place of work**	0.00	0.00	1.32	<0.00001

**Work location:** *In a municipality not in the Rhône-Alpes department*	0.00	0.00	1.58	<0.00001
**Occupation:** *Manual worker*	0.00	0.00	1.58	<0.00001
**Occupation:** *Non-manual worker*	12.50	0.29	2.11	<0.00001
**Travel mean:** *Public transport*	0.00	0.00	2.11	<0.00001
**Travel mean:** *by foot*	0.00	0.00	2.11	<0.00001
**Occupation:** *Employee*	0.00	0.00	2.11	<0.00001
**Occupation:** *Manager*	0.00	0.00	2.63	<0.00001
**Travel mean:** *Car*	0.00	0.00	3.42	<0.00001
**Work location:** *In the municipality of residence*	0.00	0.00	5.00	<0.00001
**Occupational status:** *Employed*	0.00	0.00	8.95	<0.00001

Table S3b: Description of the **profile 2** among the oldest (≥60 years)

**Variable**: *modality*	Cla/Mod [ϕ]	Mod/Cla [¥]	Global [§]	*p* value
**Satisfaction with the residential place:** *Totally agree*	48.96	94.95	59.44	<0.00001
**Perceived Air quality:** *Totally agree*	72.84	59.60	25.08	<0.00001
**Living in another place:** *No*	35.13	98.99	86.38	<0.00001
**Cardiovascular health problem:** *No*	36.58	94.95	79.57	<0.00001
**Respiratory health problem:** *No*	37.07	86.87	71.83	<0.0001
**Level of education:** *Low*	55.10	27.27	15.17	<0.001
**Knowledge about air quality:** *No*	34.85	92.93	81.73	<0.001
**Level of education:** *Baccalaureat*	87.50	7.07	2.48	<0.01
**Heating mode:** *Other*	71.43	10.10	4.33	<0.01
**Heating mode:** *Electric*	17.81	13.13	22.60	<0.01
**Satisfaction with the residential place:** *Not agree*	0.00	0.00	4.33	<0.01
**Family status:** *With partner with children*	0.00	0.00	5.57	<0.01
**Perceived Air quality:** *Not agree*	10.53	6.06	17.65	<0.001
**Knowledge about air quality:** *Yes*	8.77	5.05	17.65	<0.0001
**Respiratory health problem:** *Yes*	13.33	12.12	27.86	<0.0001
**Living in another place:** *Yes*	2.63	1.01	11.76	<0.00001

**Level of education:** *Low*	6.78	4.04	18.27	<0.00001
**Cardiovascular health problem:** *Yes*	7.58	5.05	20.43	<0.00001
**Perceived Air quality:** *Somewhat agree*	16.77	27.27	49.85	<0.00001
**Satisfaction with the residential place:** *Somewhat agree*	3.51	4.04	35.29	<0.00001

Table S3c: Description of the **profile 3** among the oldest (≥60 years)

**Variable**: *modality*	Cla/Mod ϕ	Mod/Cla ¥	Global §	*p* value
**Gender:** *man*	48.67	86.90	46.44	<0.00001
**Knowledge about air quality:** *Yes*	64.91	44.05	17.65	<0.00001
**Family status:** *With partner without children*	37.74	71.43	49.23	<0.00001
**Family status:** *With partner with children*	77.78	16.67	5.57	<0.00001
**Housing:** *House*	43.96	47.62	28.17	<0.00001
**Satisfaction with the residential place:** *Somewhat agree*	40.35	54.76	35.29	<0.0001
**Level of education:** *Intermediate*	53.13	20.24	9.91	<0.001
**Level of education:** *High*	47.62	23.81	13.00	<0.01
**Respiratory health problem:** *No*	30.17	83.33	71.83	<0.01
**Heating mode:** *Gas*	30.53	69.05	58.82	<0.05
**Perceived Air quality:** *Somewhat agreed*	31.06	59.52	49.85	<0.05
**Heating mode:** *Other*	0.00	0.00	4.33	<0.05

**Respiratory health problem:** *Yes*	15.56	16.67	27.86	<0.01
**Satisfaction with the residential place:** *Totally agree*	19.27	44.05	59.44	<0.001
**Level of education:** *Low*	8.16	4.76	15.17	<0.001
**Housing:** *Apartment*	18.97	52.38	71.83	<0.00001
**Knowledge about air quality:** *No*	17.80	55.95	81.73	<0.00001
**Family status:** *Live alone without children*	7.19	11.90	43.03	<0.00001

Table S3d: Description of the **profile 4** among the oldest (≥60 years)

**Variable**: *modality*	Cla/Mod [ɸ]	Mod/Cla [¥]	Global [§]	*p* value
**Level of education:** *Low*	79.66	45.63	18.27	<0.00001
**Gender:** *Woman*	49.13	82.52	53.56	<0.00001
**Perceived Air quality:** *Somewhat agree*	47.20	73.79	49.85	<0.00001
**Family status:** *Live alone without children*	46.04	62.14	43.03	<0.00001
**Respiratory health problem:** *Yes*	47.78	41.75	27.86	<0.001
**Satisfaction with the residential place:** *Somewhat agree*	42.98	47.57	35.29	<0.01
**Heating mode:** *Fuel oil*	55.56	19.42	11.15	<0.01
**Level of education:** *High*	85.71	5.83	2.17	<0.01
**Number years living in Lyon :** *>10 years*	33.67	98.06	92.88	<0.01
**Family status:** *With partner with children*	5.56	0.97	5.57	<0.01

**Living in another place:** *Yes*	13.16	4.85	11.76	<0.01
**Satisfaction with the residential place:** *Not agree*	0.00	0.00	4.33	<0.01
**Family status:** *With partner without children*	23.27	35.92	49.23	<0.01
**Respiratory health problem:** *No*	25.86	58.25	71.83	<0.001
**Level of education:** *Low*	6.12	2.91	15.17	<0.00001
**Level of education:** *High*	2.38	0.97	13.00	<0.00001
**Gender:** *Man*	12.00	17.48	46.44	<0.00001
**Perceived Air quality:** *Totally agree*	2.47	1.94	25.08	<0.00001

Table S3e: Description of the **profile 5 (**among the oldest (≥60 years)

**Variable**: *modality*	Cla/Mod ɸ	Mod/Cla ¥	Global §	*p* value
**Perceived Air quality:** *Not agree*	49.12	80.00	17.65	<0.00001
**Satisfaction with the residential place:** *Not agree*	92.86	37.14	4.33	<0.00001
**Living in another place:** *Yes*	52.63	57.14	11.76	<0.00001
**Level of education:** *Low*	30.61	42.86	15.17	<0.0001
**Respiratory health problem:** *Yes*	23.33	60.00	27.86	<0.0001
**Housing:** *Apartment*	14.66	97.14	71.83	<0.0001
**Cardiovascular health problem:** *Yes*	24.24	45.71	20.43	<0.001
**Family status:** *Living alone with children*	50.00	8.57	1.86	<0.05

**Level of education:** *High*	0.00	0.00	13.0	<0.01
**Cardiovascular health problem:** *No*	7.39	54.29	79.57	<0.001
**Satisfaction with the residential place:** *Somewhat agree*	4.35	20.00	49.85	<0.001
**Housing:** *House*	1.10	2.86	28.17	<0.0001
**Respiratory health problem:** *No*	6.03	40.00	71.83	<0.0001
**Perceived Air quality:** *Totally agree*	0.00	0.00	25.08	<0.0001
**Satisfaction with the residential place:** *Totally agree*	3.13	17.14	59.44	<0.00001
**Living in another place:** *No*	5.02	40.00	86.38	<0.00001

Legend: [ϕ] Percentage of individuals with the modality belonging to the profile; [¥] Percentage of individuals of the profile with the modality; [§] Percentage of the modality among the oldest.
